# Knowledge, Attitudes, and Prescribing Practices of Primary Healthcare Physicians Regarding Proton Pump Inhibitor Use in the United Arab Emirates: A Cross-Sectional Study

**DOI:** 10.7759/cureus.106308

**Published:** 2026-04-02

**Authors:** Zainab M Alameeri, Alya R Alshemeili, Omar A Alhajri, Reem M Alketbi, Zahra M Sajwani

**Affiliations:** 1 Department of Primary Healthcare, Emirates Health Services, Dubai, ARE

**Keywords:** attitudes, knowledge, practice, primary healthcare, proton pump inhibitors, united arab emirates

## Abstract

Background

Proton pump inhibitors (PPIs) are widely used to manage acid-related gastrointestinal disorders and are generally considered safe and effective. However, concerns exist about their frequent or prolonged use without clear clinical indications, which may increase the risk of adverse effects. Despite their widespread use, little is known about primary healthcare physicians’ knowledge, attitudes, and prescribing practices regarding PPIs in the United Arab Emirates. This study aimed to assess these factors among physicians within Emirates Health Services (EHS) and evaluate their awareness of potential long-term adverse effects.

Methods

An analytical cross-sectional study was conducted from February to May 2025 among primary healthcare physicians working under EHS. Data were collected using a self-administered questionnaire from a previously published validated study assessing knowledge, attitudes, and prescribing practices related to PPI use. Data were analyzed using IBM SPSS Statistics for Windows, Version 28.0 (Released 2021; IBM Corp., Armonk, NY, USA). Descriptive statistics were used to summarize the data, and a chi-square or Fisher’s exact test was used to examine associations between variables, with p < 0.05 considered statistically significant.

Results

A total of 198 physicians participated; most were aged 36-45 years (n = 88, 44.4%), female (n = 128, 64.6%), and had 11-20 years of clinical experience (n = 87, 43.9%). Overall, 166 (83.8%) demonstrated good knowledge of PPI use, and 152 (76.8%) were aware of potential adverse effects. Positive attitudes toward rational PPI use were observed in 181 (91.4%), and 187 (94.4%) reported appropriate prescribing practices based on self-report. PPI knowledge among variables tested showed that female physicians demonstrated higher levels of knowledge compared with male physicians (112 (87.5%) vs 54 (77.1%)); however, this difference did not reach the predefined threshold for statistical significance (p = 0.05). Similarly, younger physicians (≤35 years) demonstrated slightly higher ADE knowledge scores compared with older physicians; however, this association also did not reach statistical significance (p = 0.05). No statistically significant associations were found between knowledge, attitudes, and prescribing practices.

Conclusions

Primary healthcare physicians within EHS demonstrated good knowledge, positive attitudes, and generally appropriate self-reported prescribing practices regarding PPIs. However, improving rational PPI use may require not only educational interventions but also system-level strategies, such as regular review of long-term prescriptions and decision support within electronic medical records.

## Introduction

Proton pump inhibitors (PPIs) are among the most widely prescribed drugs for managing acid-related gastrointestinal diseases [1] such as GERD, peptic ulcers, and Zollinger-Ellison syndrome [2]. These drugs reduce gastric acid secretion by irreversibly inhibiting the hydrogen-potassium ATPase enzyme (proton pump) in the stomach’s parietal cells [3]. PPIs significantly reduce gastric acid secretion, promoting mucosal healing and lowering the risk of complications such as bleeding ulcers [4]. Commonly prescribed PPIs include esomeprazole, lansoprazole, omeprazole, dexlansoprazole, pantoprazole, and rabeprazole [5]. PPIs have been approved as over-the-counter treatments in several countries, enabling patients to self-medicate for dyspepsia and other gastric conditions [3].

While PPIs are effective and generally safe, their appropriate use requires healthcare professionals, particularly those in primary care, to thoroughly understand their indications, contraindications, and potential long-term effects [2]. A study in the United Arab Emirates (UAE) indicates that approximately 79% of individuals use PPIs based on physicians’ recommendations [6]. However, two studies conducted in the Gulf Cooperation Council (GCC) region suggest variability in physicians’ knowledge and prescribing patterns. In Kuwait, 74% of physicians were aware of PPI-related risks and adjusted their prescribing practices accordingly, highlighting the need for evidence-based education to optimize PPI use [7]. In Saudi Arabia, similar trends were observed. Although physicians generally demonstrated favorable attitudes toward PPIs, many lacked sufficient knowledge, highlighting the need for regular professional development to mitigate inappropriate prescribing [1].

Despite their widespread use, PPIs are frequently prescribed for extended periods without clear indications, increasing the risk of unnecessary exposure to potential adverse effects. In a Saudi Arabian study, only 55.1% of physicians identified all potential adverse effects of prolonged therapy [8]. Furthermore, long-term PPI use has been linked to complications, including vitamin and mineral malabsorption [9], increased risk of community-acquired pneumonia [10], bone fractures [11], kidney impairment [12], *Clostridium difficile* infections [13], dementia [14], and cardiovascular events [15,16]. Moreover, a recent study found that 62% of physicians expressed concerns about PPIs’ potential adverse effects, yet prescribing behaviors often deviated from best-practice guidelines [7].

In primary healthcare settings, physicians are pivotal in ensuring PPIs are used rationally, balancing the therapeutic benefits with potential risks. Physicians’ knowledge, attitudes, and practices (KAP) regarding PPI use significantly influence prescribing behavior and patient outcomes. Although guidelines provide the fundamental framework for appropriate PPI use, one study suggests that prescribing patterns are influenced by physicians’ educational background, clinical experience, personal beliefs, and the pharmaceutical industry’s marketing and promotion strategies [17].

Despite the widespread availability of PPIs and the high prevalence of acid-related disorders in the UAE, data on primary healthcare physicians’ knowledge, attitudes, and prescribing behaviors remain limited. Understanding these factors is essential for improving prescribing practices, minimizing unnecessary long-term use, and mitigating associated risks.

This cross-sectional study aims to assess the KAP of primary healthcare physicians within Emirates Health Services (EHS) regarding PPI use. A secondary objective is to evaluate physicians’ knowledge of PPI-related adverse effects. Findings from this study will help identify gaps in current clinical practice, guide targeted educational interventions, and support policy development to optimize PPI prescribing within EHS, ultimately enhancing patient care and clinical outcomes.

## Materials and methods

Study design and setting

This analytical cross-sectional study was conducted between February and May 2025 among physicians working in primary healthcare facilities under EHS that provide public healthcare services across the Northern Emirates (Sharjah, Ajman, Umm Al Quwain, Ras Al Khaimah, and Fujairah, as well as selected facilities in Dubai). Ethical approval was obtained from the Ministry of Health and Prevention Research Ethics Committee (approval MOHAP/DXB-REC/M.M.M/No. 47/2025).

Participants

The study population included licensed primary healthcare physicians and senior family medicine residents working in EHS primary healthcare facilities. Dentists, public health physicians, physicians working in secondary or tertiary care, junior residents, and interns within EHS were excluded. Participation was voluntary, and electronic informed consent was obtained from all participants.

Sample size and sampling

The minimum required sample size was 198 and calculated using the Raosoft online sample size calculator (Raosoft, Inc., Seattle, WA, USA), assuming an expected prevalence of 50%, a 95% confidence level, and a 5% margin of error. Of the 404 eligible EHS primary healthcare physicians reported in the 2024 EHS Open Data Source, 198 physicians completed the questionnaire, corresponding to a response rate of 49%. Participants were recruited using convenience sampling. Participation was voluntary and anonymous, which may have encouraged candid responses and reduced response bias. Although convenience sampling may introduce selection bias and nonresponse bias, the survey’s wide distribution across geographic locations was intended to enhance representativeness within the EHS primary healthcare district.

Data collection instrument

A self-administered questionnaire adapted from a previously validated survey by Luo et al. was used [18]. In the original study, the instrument demonstrated acceptable internal consistency with a Cronbach’s alpha coefficient of 0.78. Contextual modifications were limited to the demographic domain to ensure alignment with the UAE primary healthcare setting. The questionnaire comprised four domains: demographics, knowledge, prescribing attitudes, and prescribing behaviors related to PPI use.

The demographics domain included participants’ gender, age, years of clinical experience, highest attained medical qualification, emirate of current workplace, specialty, and current job title. The list of questionnaire statements is presented in Table 1. The questionnaire was pilot-tested prior to data collection to ensure clarity and feasibility.

**Table 1:** Questionnaire statements assessing physicians’ knowledge, attitudes, and prescribing practices regarding PPIs Adapted from Luo et al. (2019) [18], with modifications to the demographic domain EHS, Emirates Health Services; PPI, proton pump inhibitor

Domain	Item	Statement	Response options
Knowledge	1	Is PPI an inactive prodrug?	Yes/No
	2	Do PPIs include omeprazole, pantoprazole, rabeprazole, esomeprazole, lansoprazole, etc.?	Yes/No
	3	Do PPIs cure acid-related diseases by suppressing hydrochloric acid secretion?	Yes/No
	4	Can PPIs be used to prevent stress ulcers?	Yes/No
	5	Can PPIs be used to treat acute pancreatitis?	Yes/No
	6	Does omeprazole have the largest individual difference compared with other PPIs?	Yes/No
	7	Does omeprazole have the largest interaction compared to other PPIs?	Yes/No
	8	Does omeprazole have the longest acid inhibition time compared to other PPIs?	Yes/No
	9	Should omeprazole be selected for pediatric patients?	Yes/No
	10	Should rabeprazole be selected for pregnant patients?	Yes/No
	11	Do you think the more expensive or newer PPI will produce a better and safer effect?	Yes/No
	12	Is PPI usually available as enteric-coated capsules or tablets?	Yes/No
	13	Should PPI usually be taken at breakfast?	Yes/No
	14	Should PPI be taken after meals?	Yes/No
	15	Should PPI be swallowed whole?	Yes/No
	16	Is it advisable to increase the dose frequency rather than have a single dose to improve the effect?	Yes/No
	17	Should patients take a PPI for only seven days in the *Helicobacter pylori* eradication therapy?	Yes/No
	18	Does PPI treatment of gastric ulcers take two to four weeks?	Yes/No
	19	Is the duration of PPI prophylaxis until no high-risk factors are present or able to tolerate enteral feeding?	Yes/No
	20	Do you think long-term use of PPI may cause adverse reactions such as osteoporosis?	Yes/No
	21	Do you think long-term use of PPI may cause adverse reactions such as pneumonia?	Yes/No
	22	Do you think long-term use of PPI may cause adverse reactions such as *Clostridium difficile*?	Yes/No
	23	Do you think long-term use of PPI may cause adverse reactions such as hip fractures?	Yes/No
Attitude	1	Overuse of PPI is commonly present in EHS primary healthcare facilities.	Yes/No
	2	The main cause of PPI overuse is doctors’ or patients’ abuse of PPIs.	Yes/No
	3	The main purpose of PPI overuse is stress ulcer prophylaxis.	Yes/No
	4	Overuse of PPI will cause an increase in adverse drug reactions and medical costs.	Yes/No
	5	Is it necessary to carry out large-scale education on the rational use of PPI for medical staff and the public?	Yes/No
	6	Use of PPI for a short duration does not cause significant side effects.	Yes/No
Practice	1	Do you prescribe PPI for abdominal pain?	Yes/No
	2	Do you prescribe PPI for flatulence (ventosity)?	Yes/No
	3	Do you prescribe PPI for nausea?	Yes/No
	4	Do you prescribe PPI for vomiting?	Yes/No
	5	Do you prescribe PPI for acid reflux?	Yes/No
	6	Do you prescribe PPI for dyspepsia?	Yes/No

Data collection procedure

Following ethical approval, the questionnaire and participant informed consent were digitalized through the EHS Data-Hub platform. The questionnaire was distributed electronically to primary healthcare physicians via institutional email and SMS. An initial pilot phase was conducted, after which the finalized questionnaire was disseminated for data collection. Data collection took place between March and May 2025. The questionnaire was self-administered, and participants provided electronic consent before accessing the survey. Upon submission, responses were automatically captured and stored in a secure, password-protected Excel database within the EHS Data-Hub platform, accessible only to the research team.

Variable construction and scoring

The questionnaire assessed knowledge, prescribing attitudes, and prescribing behaviors regarding PPI use. Knowledge and prescribing behaviors items employed dichotomous (Yes/No) responses. Prescribing attitudes items also used dichotomous (Yes/No) responses, except for one item assessing the main cause of PPI overuse with three response options (doctor, patient, or both).

The knowledge domain consisted of 23 items assessing physicians’ knowledge regarding PPIs. Each correct response was assigned a score of one, and incorrect responses were assigned zero. The total knowledge score ranged from 0 to 23 and was categorized into poor knowledge (0-11) and good knowledge (12-23). In addition, four knowledge items specifically assessed physicians’ awareness of potential adverse drug effects (ADEs) associated with long-term PPI use (items 20-23 in the questionnaire). An ADE knowledge score was calculated separately based on these four items. Scores ranged from 0-4 and were categorized as poor ADE knowledge (0-2) and good ADE knowledge (3-4). The attitude domain included six items evaluating physicians’ attitudes toward PPI use in the primary care setting. Each positive response was assigned one point, with total scores ranging from 0 to 6. Scores were categorized as poor attitude (0-3) and good attitude (4-6). The practice domain comprised six items assessing self-reported PPI prescribing behaviors. Each appropriate practice response was assigned one point, giving a total score ranging from 0 to 6. Scores were categorized as poor practice (0-3) and good practice (4-6). Most questionnaire items used dichotomous (Yes/No) response options.

The score cutoffs used to categorize KAP levels were based on the scoring framework applied in the original validated questionnaire.

Statistical analysis

Data were analyzed using IBM SPSS Statistics for Windows, Version 28.0 (Released 2021; IBM Corp., Armonk, NY, USA). Incomplete responses were excluded during preliminary data cleaning. Participants’ ages were grouped into ≤35, 36-45, and ≥46 years, and other demographic variables were categorized as appropriate for analysis. Descriptive statistics were used to summarize participant characteristics and KAP variables, presented as frequencies and percentages. Associations between categorical variables were examined using Pearson’s chi-square test where appropriate. For variables with sparse cell counts and expected frequencies below 5 across multiple categories, inferential testing was not performed, and findings were reported descriptively. Fisher’s exact test was used where appropriate. A p-value of < 0.05 was considered statistically significant.

## Results

Characteristics of participants

A total of 198 primary healthcare physicians were included in the final analysis, following the exclusion of two incomplete responses. Participant characteristics are presented in Table 2. The majority were aged 36-45 years, 88 (44.4%). Most participants were female, 128 (64.6%). Clinical experience was substantial across the sample, with 87 (43.9%) reporting 11-20 years, and 49 (24.7%) reporting ≥21 years of practice. By specialty, 92 (46.5%) family physicians and 90 (45.5%) general practitioners constituted the majority.

**Table 2:** Demographic and professional characteristics of participating physicians (N = 198)

Characteristic	Category	n (%)
Age group	≤35 years	36 (18.2%)
	36-45 years	88 (44.4%)
	46 years and above	74 (37.4%)
Gender	Female	128 (64.6%)
	Male	70 (35.4%)
Clinical experience	≤5 years	18 (9.1%)
	6-10 years	44 (22.2%)
	11-20 years	87 (43.9%)
	≥21 years	49 (24.7%)
Qualifications	MBBS/MD	82 (41.4%)
	Postgraduate diploma	17 (8.5%)
	Master’s degree	33 (16.7%)
	Board certification/fellowship	66 (33.3%)
Emirate of workplace	Sharjah	64 (32.3%)
	Ras Al-Khaimah	40 (20.2%)
	Fujairah	36 (18.2%)
	Ajman	26 (13.1%)
	Dubai	22 (11.1%)
	Umm Al Quwain	10 (5.1%)
Specialty	Family physician	92 (46.5%)
	General practitioner	90 (45.5%)
	Senior family medicine resident	9 (4.5%)
	Other	7 (3.5%)
Job title	General practitioner	121 (61.1%)
	Family medicine specialist	34 (17.2%)
	Family medicine consultant	23 (11.6%)
	Family medicine resident and other	20 (10.1%)

Knowledge of participants regarding PPI use

Overall knowledge of PPI use was high among physicians. A total of 166 (83.8%) demonstrated good knowledge, while 32 (16.2%) showed poor knowledge. The mean knowledge score among participants (n = 198) was 15 ± 3 (range: 7-22). Awareness of PPI-related adverse effects (ADEs) was generally high, with 152 (76.8%) of physicians demonstrating good ADE knowledge and 46 (23.2%) categorized as having poor ADE knowledge. The mean ADE knowledge score among participants (N = 198) was 3 ± 1 (range: 0-4). A summary of physicians’ knowledge, attitudes, and prescribing practice scores is presented in Table 3.

**Table 3:** Summary of physicians’ knowledge, attitudes, and prescribing practices regarding PPIs (N = 198) PPI, proton pump inhibitor

Domain	Score range	Mean ± SD	Poor, n (%)	Good, n (%)
Knowledge	0-23	15 ± 3	32 (16.2%)	166 (83.8%)
Attitude	0-6	4 ± 1	17 (8.6%)	181 (91.4%)
Practice	0-6	5 ± 1	11 (5.6%)	187 (94.4%)

In assessing factors associated with physicians’ awareness, gender was the only variable that showed a trend toward higher overall knowledge levels. Female physicians demonstrated slightly higher levels of knowledge compared with male physicians (112 (87.5%) vs 54 (77.1%)); however, this difference did not reach the predefined threshold for statistical significance (p = 0.05) (Table 4).

**Table 4:** Association between physicians’ knowledge level regarding PPIs and demographic characteristics (N = 198) p-Values were calculated using Pearson’s chi-square test where appropriate. For variables with sparse cell counts and expected frequencies below 5, inferential testing was not performed; these data are presented descriptively. PPI, proton pump inhibitor

Variable	Category	Poor knowledge, n (%)	Good knowledge, n (%)	p-Value
Gender	Male	16 (22.9%)	54 (77.1%)	0.05
	Female	16 (12.5%)	112 (87.5%)	
Age group	≤35 years	3 (8.3%)	33 (91.7%)	0.27
	36-45 years	14 (15.9%)	74 (84.1%)	
	46 years and above	15 (20.3%)	59 (79.7%)	
Clinical experience	≤5 years	3 (16.7%)	15 (83.3%)	-
	6-10 years	6 (13.6%)	38 (86.4%)	
	11-20 years	13 (14.9%)	74 (85.1%)	
	≥21 years	10 (20.4%)	39 (79.6%)	
Qualifications	MBBS/MD	10 (12.2%)	72 (87.8%)	-
	Postgraduate diploma	4 (23.5%)	13 (76.5%)	
	Master’s degree	8 (24.2%)	25 (75.8%)	
	Board certification/fellowship	10 (15.2%)	56 (84.8%)	
Emirate of workplace	Ajman	3 (11.5%)	23 (88.5%)	-
	Dubai	3 (13.6%)	19 (86.4%)	
	Fujairah	10 (27.8%)	26 (72.2%)	
	Ras Al-Khaimah	7 (17.5%)	33 (82.5%)	
	Sharjah	8 (12.5%)	56 (87.5%)	
	Umm Al Quwain	1 (10.0%)	9 (90.0%)	
Specialty	General practitioner	14 (15.6%)	76 (84.4%)	-
	Senior family medicine resident	0 (0.0%)	9 (100.0%)	
	Family physician	17 (18.5%)	75 (81.5%)	
	Other	1 (14.3%)	6 (85.7%)	
Job title	General practitioner	19 (15.7%)	102 (84.3%)	-
	Family medicine resident and other	1 (5.0%)	19 (95.0%)	
	Family medicine specialist	7 (20.6%)	27 (79.4%)	
	Family medicine consultant	5 (21.7%)	18 (78.3%)	

Regarding knowledge of adverse effects (ADEs), younger physicians (≤35 years) demonstrated slightly higher ADE knowledge scores compared with older physicians; however, this difference did not reach the predefined threshold for statistical significance (p = 0.05) (Table 5).

**Table 5:** Association between physicians’ knowledge of PPI-related ADEs and demographic characteristics (N = 198) p-Values were calculated using Pearson’s chi-square test where appropriate. For variables with sparse cell counts and expected frequencies below 5, inferential testing was not performed; these data are presented descriptively. ADE, adverse drug effect; PPI, proton pump inhibitor

Variable	Category	Poor ADE knowledge, n (%)	Good ADE knowledge, n (%)	p-Value
Gender	Male	16 (22.9%)	54 (77.1%)	0.92
	Female	30 (23.4%)	98 (76.6%)	
Age group	≤35 years	4 (11.1%)	32 (88.9%)	0.05
	36-45 years	19 (21.6%)	69 (78.4%)	
	46 years and above	23 (31.1%)	51 (68.9%)	
Clinical experience	≤5 years	5 (27.8%)	13 (72.2%)	-
	6-10 years	6 (13.6%)	38 (86.4%)	
	11-20 years	19 (21.8%)	68 (78.2%)	
	≥21 years	16 (32.7%)	33 (67.3%)	
Qualifications	MBBS/MD	17 (20.7%)	65 (79.3%)	-
	Postgraduate diploma	3 (17.6%)	14 (82.4%)	
	Master’s degree	11 (33.3%)	22 (66.7%)	
	Board certification/fellowship	15 (22.7%)	51 (77.3%)	
Emirate of workplace	Ajman	9 (34.6%)	17 (65.4%)	-
	Dubai	5 (22.7%)	17 (77.3%)	
	Fujairah	9 (25.0%)	27 (75.0%)	
	Ras Al-Khaimah	6 (15.0%)	34 (85.0%)	
	Sharjah	13 (20.3%)	51 (79.7%)	
	Umm Al Quwain	4 (40.0%)	6 (60.0%)	
Specialty	General practitioner	21 (23.3%)	69 (76.7%)	-
	Senior family medicine resident	0 (0.0%)	9 (100.0%)	
	Family physician	22 (23.9%)	70 (76.1%)	
	Other	3 (42.9%)	4 (57.1%)	
Job title	General practitioner	35 (28.9%)	86 (71.1%)	-
	Family medicine resident and other	2 (10.0%)	18 (90.0%)	
	Family medicine specialist	6 (17.6%)	28 (82.4%)	
	Family medicine consultant	3 (13.0%)	20 (87.0%)	

No other tested demographic variables were significantly associated with either PPI knowledge or ADE knowledge (p > 0.05).

Attitude of participants regarding PPI use

Physicians generally demonstrated positive attitudes toward rational PPI use. Overall, 181 (91.4%) of participants showed good attitude scores, while 17 (8.6%) were categorized as having poor attitude scores (Table 3).

Participants’ prescribing practices toward PPI use

Self-reported prescribing behavior was generally appropriate, with 187 (94.4%) of physicians categorized as having good practice scores, and 11 (5.6%) classified as having poor practice scores (Table 3).

Association between KAP toward PPI use

Fisher’s exact test showed that no statistically significant associations were observed between physicians’ practice and their knowledge (p = 1.00) or attitude (p = 0.60) toward PPI use. Similarly, there was no significant relationship between knowledge and attitude (p = 0.74). These findings indicate that although most participants demonstrated good knowledge, positive attitudes, and appropriate practice toward PPIs, these domains were not statistically associated with one another.

## Discussion

Prior assessments of KAP regarding PPI use among primary healthcare physicians in the UAE have been limited. This cross-sectional study represents one of the first comprehensive evaluations of PPI-related KAP among primary healthcare physicians working within EHS. The findings revealed that 166 physicians (83.8%) demonstrated high overall knowledge, 152 (76.8%) were aware of PPI-related adverse effects, 181 (91.4%) exhibited positive attitudes toward rational PPI use, and 187 (94.4%) reported generally appropriate prescribing practices. The high levels of self-reported appropriate prescribing practices observed in this study may partly reflect social desirability bias inherent to self-reported surveys. Nevertheless, these findings suggest a generally favorable self-reported KAP profile among physicians working in EHS primary healthcare settings. The overall knowledge score was higher among female physicians compared with male physicians (112 (87.5%) vs 54 (77.1%)); however, this difference did not reach the predefined threshold for statistical significance (p = 0.05). Similarly, awareness of PPI adverse effects was somewhat higher among physicians aged ≤35 years compared with older age groups; however, this difference did not reach the predefined threshold for statistical significance (p = 0.05). These observations should therefore be interpreted cautiously but may reflect differences in training exposure, engagement with continuing professional development activities, or greater familiarity with updated prescribing guidelines among younger physicians.

When compared with studies from the GCC and Asian countries, physicians in this UAE cohort demonstrated markedly higher knowledge levels, while attitudes and prescribing practices were generally comparable. For instance, an Omani study reported good knowledge in only 20.9% of primary healthcare physicians, contrasted with high levels of positive attitudes (96.7%) and prescribing practices (96.1%) [19]. Likewise, Saudi Arabian data highlighted gaps in physicians’ knowledge and frequent prescribing of PPI for nonevidence-based indications or extended durations [1]. Moreover, poor PPI knowledge in China was linked to younger healthcare professionals, individuals with lower educational levels, females, and those who are employed in a private hospital [18]. However, Luo et al. [18] studied multiple healthcare professionals, whereas the present study included primary healthcare physicians only. Collectively, these findings position EHS physicians favorably within the regional knowledge spectrum, though high self-reported attitudes and practices across GCC studies do not necessarily translate into optimal long-term PPI use.

Notably, no significant correlations were identified between KAP domains (p > 0.05), suggesting that prescribing patterns may be influenced by factors beyond knowledge and attitude alone. These factors may include habitual prescribing patterns, patient expectations for symptomatic relief, and automated prescription renewals as described in broader PPI stewardship literature. Higher awareness does not necessarily translate into deprescribing, particularly for prolonged therapy without clear clinical indications, such as functional dyspepsia or extended stress ulcer prophylaxis. Although this study demonstrates superior KAP scores compared to several regional and Asian studies, concerns regarding long-term PPI exposure risks, including fractures, infections, and renal impairment, remain clinically relevant. These findings underscore that physician education alone is insufficient and should be complemented by system-level stewardship.

Female physicians and younger participants demonstrated slightly higher knowledge levels in this study; however, these differences did not reach statistical significance and should therefore be interpreted cautiously. Further studies could explore whether peer learning or targeted educational strategies involving these groups may contribute to improving awareness of rational PPI use. Additionally, embedding standardized PPI algorithms in electronic medical records (EMRs) with mandatory fields for indication, planned duration, automated deprescribing prompts alongside, and enhanced patient counseling on definite therapy and lifestyle measures could better translate knowledge into consistent behavior and reduce chronic overuse driven by patient demand. This approach is aligned with international PPI prescribing patterns and utilization studies that advocate EMR-based decision support, periodic review of indication and dose, and incorporation of pharmacists into medication review to limit long-term inappropriate use.

Policy recommendations include establishing a formal PPI stewardship framework across EHS, harmonizing primary/secondary/tertiary care guidelines with explicit initiation criteria (e.g., confirmed GERD or ulcers), step-down protocols, and review intervals (e.g., every three to six months for long-term use), supported by regular audits and feedback. Such a framework would address the same overuse patterns reported in Saudi and Omani settings, where PPIs are frequently prescribed for extended durations or for weak indications, despite generally positive attitudes and acceptable reported practices [1,19].

Future research should expand KAP assessment to include hospital-based physicians and pharmacists using mixed-methods approaches, such as qualitative interviews to explore workload barriers and institutional norms to deprescribing. Targeted continuing professional development for lower-knowledge subgroups and physicians aged 46 years and above should prioritize deprescribing algorithms and individualized risk-benefit assessment. Additionally, intervention studies evaluating EMR decision support tools, routine audits, and pharmacist-involved stewardship are needed to assess their impact on prescribing quality indicators and patient outcomes. Multicenter or multicountry studies across the Gulf region would help clarify how differences in training, guidelines, and health system structure contribute to the observed variation and knowledge percentages.

Limitations

This study has limitations that should be acknowledged. First, the sample was restricted to primary healthcare physicians working within EHS and did not include hospital-based physicians or other healthcare professionals such as pharmacists. Consequently, the findings may not be generalized to the broader healthcare workforce involved in PPI prescribing and monitoring. Given that many PPIs are initiated in hospital settings or continued under specialist care, excluding these groups may underestimate important contributors to long-term or inappropriate use. Second, convenience sampling was used to recruit participants, which may introduce selection bias. Physicians who chose to participate may have been more interested in rational pharmacotherapy or more aware of PPI prescribing guidelines, potentially leading to an overestimation of knowledge levels and appropriate prescribing practices. Additionally, as participation was voluntary, nonresponse bias cannot be excluded, and the perspectives of physicians who did not respond may differ from those who participated. Third, prescribing practices were assessed using self-reported responses rather than objective prescribing records, which may introduce reporting bias and social desirability bias. Recall bias may also have affected participants’ responses. Finally, the cross-sectional design precludes causal inference between knowledge, attitudes, and prescribing practices. The absence of EMRs or pharmacy dispensing data limits the ability to validate self-reported prescribing behavior. No multivariable analysis was performed to adjust for potential confounders.

## Conclusions

Primary healthcare physicians within EHS generally demonstrated good knowledge levels, positive attitudes, and self-reported appropriate prescribing practices regarding PPI use. However, the absence of a significant association between knowledge, attitudes, and prescribing practices suggests that clinical decision-making may be influenced by factors beyond knowledge alone. These findings suggest that improving rational PPI use requires not only educational interventions but also system-level strategies, such as regular review of long-term prescriptions and integration of decision support tools within EMRs. Future studies using objective prescribing data are needed to better evaluate actual prescribing practices and inform targeted stewardship interventions.
